# Phytochemicals Rosmarinic Acid, Ampelopsin, and Amorfrutin-A Can Modulate Age-Related Phenotype of Serially Passaged Human Skin Fibroblasts *in vitro*

**DOI:** 10.3389/fgene.2019.00081

**Published:** 2019-02-21

**Authors:** Lakshman Sodagam, Anna Lewinska, Ewa Kwasniewicz, Sofiya Kokhanovska, Maciej Wnuk, Karsten Siems, Suresh I. S. Rattan

**Affiliations:** ^1^ Laboratory of Cellular Ageing, Department of Molecular Biology and Genetics, Aarhus University, Aarhus, Denmark; ^2^ Department of Cell Biochemistry, University of Rzeszow, Rzeszow, Poland; ^3^ Department of Genetics, University of Rzeszow, Rzeszow, Poland; ^4^ AnalytiCon Discovery GmbH, Potsdam, Germany

**Keywords:** senescence, lifespan, health-span, aging, hormesis, hormetin

## Abstract

One of the aims of the EU-funded Research and Innovation Action (RIA), titled “Ageing with Elegans” (AwE) is to enhance better understanding of the factors causing health and disease in aging and develop evidence-based preventive, diagnostic, therapeutic, and other strategies. The work package-5 of this project is focused on testing the effects of phytochemicals of natural and synthetic origin on aging, longevity, and health of human cells *in vitro*, after the initial screening using the animal model systems of nematodes and rats and mice. Accordingly, the first series of three compounds, rosmarinic acid (ROSM), ampelopsin (AMPEL), and amorfrutin-A (AMOR), were selected to test for their short-term and long-term effects on human skin fibroblasts undergoing aging and senescence *in vitro.* The lifelong modulatory effects of these compounds were tested individually at two doses (0.5 and 1.0 μM), selected after a short-term dose response check of a 20,000-fold range (0.01–200 μM). The results show that these compounds do have some beneficial effects in terms of supporting the long-term lifelong growth and enhanced stress tolerance of serially passaged cells. These effects seem to be achieved by reducing the extent of loss of telomeres, of 5-methyl-cytosine (5-mC) and of 5-hydroxymethyl-cytosine (5-hmC), by reducing the accumulation of oxidative DNA damage product 8-OHdG. There is also some indication that these compounds induce at least one of the stress responses in terms of the increased synthesis of heat shock protein Hsp70. Thus, these phytochemicals may be potential hormetins, which bring about their health beneficial effects by the phenomenon of mild stress-induced hormesis.

## Introduction

Modulating aging for achieving healthy old age and extended health span is one of the most popular and challenging themes in modern biogerontology (Rattan, 2018). Numerous governmental and private R&D programs are ongoing worldwide to address this issue. Healthy aging has been selected by the European Union (EU) policy makers as an important priority, and it figures prominently in the Horizon-2020 program launching several research and innovation actions (RIA). One of these actions is titled “Understanding health, ageing and disease: determinants, risk factors and pathways”, in the work program on “Personalising healthcare”.^1^ Within this framework, one project entitled “Ageing with elegans” (AwE),^2^ aims for better understanding of the factors causing health and disease in aging and develop evidence-based preventive, diagnostic, therapeutic, and other strategies (Luyten et al., 2016). One of the work packages (WP-5) of this project is focused on testing the effects of phytochemicals of natural and synthetic origin on aging, longevity, and health of human cells *in vitro*. The test compounds for human application are selected after their initial screening through other model systems, including the nematode *Caenorhabditis elegans* and rats and mice (Luyten et al., 2016).

For human cell culture-based studies, the so-called Hayflick system of normal human fibroblasts undergoing replicative senescence *in vitro* was used (Hayflick and Moorhead, 1961; Hayflick, 1965; Rattan and Hayflick, 2016). The Hayflick system is comprised of serially passaged normal diploid differentiated cells, which undergo progressive and intrinsic age-related changes resulting in the culmination of cell proliferation, also known as the replicative senescence. Hundreds of changes at the structural, physiological, biochemical, and molecular levels have been described for this model system of cellular aging and replicative senescence, most of which are also applicable *in vivo* (Campisi and d’Adda di Fagagna, 2007; Rattan, 2008; Rattan and Hayflick, 2016; Yanai and Fraifeld, 2018). Senescent cells are also one of the hallmarks of aging of the organisms (Lopez-Otin et al., 2013). Therefore, during the last 50 years, this Hayflick system has been a widely used experimental model system for studies on cellular aging and replicative senescence, and has resulted both in unraveling the molecular mechanisms of cellular aging and in screening potential aging-modulatory compounds (Campisi and d’Adda di Fagagna, 2007; Rattan, 2008; Rattan and Hayflick, 2016; Yanai and Fraifeld 2018).

**Figure 1:**
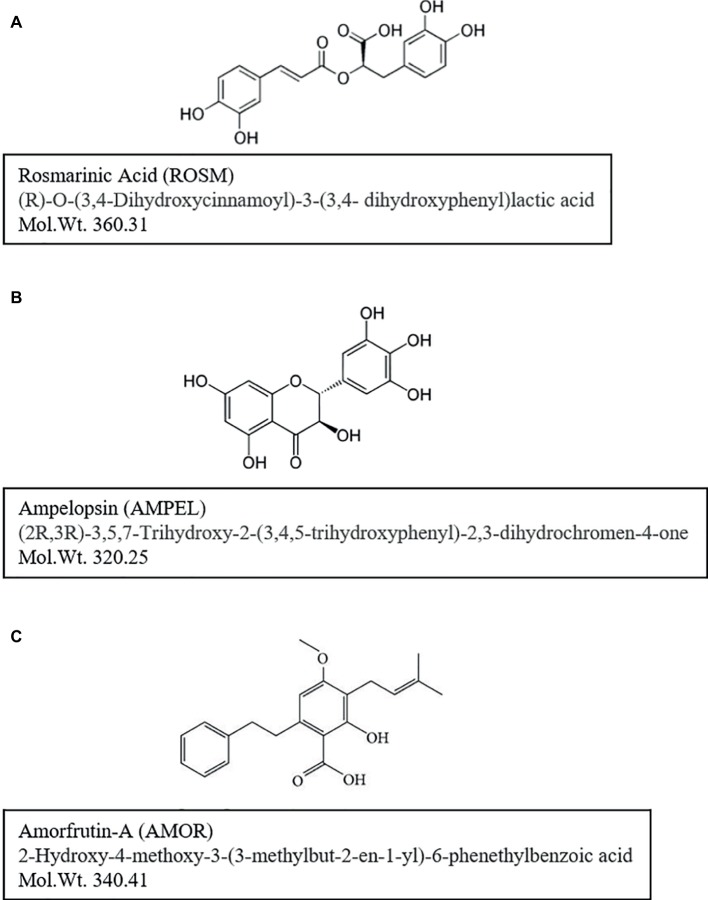
Chemical structures and molecular weights of test compounds: **(A)** rosmarinic acid (ROSM), **(B)** ampelopsin (AMPEL), and **(C)** amorfrutin-A (AMOR). Further information on the identity and purity confirmation of compounds NMR and HPLC-MS-ELSD is available on file at the AnalytiCon Discovery, Germany.

The first series of three compounds, rosmarinic acid (ROSM), ampelopsin (AMPEL), and amorfrutin-A (AMOR), were selected to test for their short-term and long-term effects on normal diploid human skin fibroblasts undergoing aging and senescence. A brief description of the three compounds and their background with respect to biological activities is given below (Figure 1).

ROSM is an ester of caffeic acid and 3,4-dihydroxyphenyllactic acid (Figure 1A). As reviewed by Petersen and Simmonds (2003) and Amoah et al. (2016), ROSM is an active ingredient in several spices and herbs like Rosemary, Perilla, Mentha, Saliva, and others. It is commonly found in species of the subfamily *Nepetoideae* of the *Lamiaceae* in *Boraginaceae*, and a few other plant families including some fern and hornwort species. ROSM has a number of interesting biological activities, such as being antiviral, antibacterial, anti-inflammatory, and an antioxidant. For example, ROSM has been reported to protect human dopaminergic neuronal cells against hydrogen peroxide-induced apoptosis (Lee et al., 2008a,b). Furthermore, LPS-induced production of monocyte chemoattractant protein-1 (MCP-1) and macrophage inflammatory protein-1alpha (MIP-1alpha) *via* the MAPK pathway is down regulated by ROSM in mouse dendritic cells (Kim et al., 2008). ROSM also mitigates general symptoms of atopic dermatitis in humans and may have potential anti-cancer effects as well (Lee et al., 2008a,b). With respect to aging and longevity, ROSM has been shown to extend lifespan and thermotolerance in *C. elegans* (Pietsch et al., 2011).

AMPEL, (2R,3R)-3,5,7-trihydroxy-2-(3,4,5-trihydroxyphenyl)-2,3-dihydrochromen-4-one; Figure 1B) is one of the major flavonoids present in the Chinese herb *Ampelopsis grossedentata*. It is also known as dihydromyricetin and has a similar structure to myricetin (3,5,7-trihydroxy-2-(3,4,5-trihydroxyphenyl)-4-chromenone), a naturally occurring flavonoid found in fruits, vegetables, herbs, grapes, and berries (Ni et al., 2012). AMPEL has been reported to have various biological activities, including anti-oxidative, hypoglycemic, anti-angiogenic, and anti-tumoric activities (Ni et al., 2012). AMPEL is also reported to attenuate d-galactose-induced brain aging in rats, through miR-34a-mediated SIRT1/mTOR signal pathway (Kou et al., 2016). It is also shown that the atrophy of skeletal muscle from those rats is reduced, by activating AMPK/SIRT1/PGC-1alpha signaling cascade (Kou et al., 2017).

AMOR, (2-hydroxy-4-methoxy-3-(3-methylbut-2-en1-yl)-6-phenethylbenzoic acid; Figure 1C) is an isoprenoid-substituted benzoic acid product isolated from the edible parts of two legumes, *Glycyrrhiza foetida* and *Amorpha fruticosa* (Weidner et al., 2012). It has been studied for its anti-diabetic effects achieved by binding to and activating PPAR-gamma, resulting in altered gene expression and physiological profiles that are significantly different from activation by other synthetic PPAR-gamma drugs (Weidner et al., 2012, 2013). Furthermore, AMOR is reported to exert anticancer effects by inhibiting STAT3 activation in cervical cancer cells (Mi et al., 2017).

Here, we present the results of our studies on the effects of ROSM, AMPEL, and AMOR, tested individually, on the survival, growth rates, longevity, aging markers, and stress tolerance of serially passaged normal human skin fibroblasts during their replicative lifespan *in vitro*. Our results indicate that all three compounds have some aging-modulatory and health-promoting effects to varying extents.

## Materials and Methods

### Cell Culture

Normal human skin fibroblast cell strain, here referred to as PCS cells, used in this study was the primary cultures established from a skin biopsy from the abdomen region of a healthy 38-year-old Caucasian woman, purchased from ATCC (Catalog No: PCS-201-012 Lot No. 58732338) As described previously (Sodagam et al., 2017): “cells were cultured in plastic tissue culture flasks (T_75_ cm^2^) in an incubator at 37°C in a humidified atmosphere in the presence of 5% CO_2_ using Dulbecco’s Modified Eagle’s Medium (DMEM; Lonza, USA), supplemented with 10% fetal bovine serum (Lonza, USA), and a mix solution of antibiotic and antimycotic agents (100 U/ml penicillin, 0.1 mg/ml streptomycin, and 0.25 mg/ml amphotericin B; Lonza, USA). This is referred to as the complete culture medium (CCM). For sub-culturing, cells were trypsinized and seeded into new culture flasks at 1:2 or 1:4 ratio, adding one or two passages (p) to the culture age, respectively. For full-term longevity studies, cells were serially passaged each time the cultures became more than 95% confluent. Cell numbers were monitored by using a Coulter counter and were used to calculate the exact number of population doublings (PD) and cumulative PD levels (CPDL) achieved *in vitro*. Microscopic-photographic records of cells were kept either as phase-contrast pictures of live cells or of methanol-fixed and Giemsa stained cells, using a Zeiss inverted microscope”.

### Phytochemicals

ROSM, AMPEL, and AMOR were provided by the collaborative partner in this project, AnalytiCon Discovery (Germany). ROSM (mol. wt. 360.31) was purchased from Carbosynth, Berkshire, UK (lot number FR023101501), and its identity and purity (100%) were confirmed by NMR and HPLC-MS-ELSD at AnalytiCon Discovery. AMPEL (mol. wt. 320.25) was purchased from TransMIT, Gießen, Germany, and its identity and purity (100%) were confirmed by NMR and HPLC-MS. AMOR (mol. wt. 340.41) was isolated from roots of *Glycyrrhiza foetida* (Friedrich Nature Discovery, Euskirchen, Germany) by reverse phase chromatography (batch ID C-0595-D10), and its identity and purity (100%) was confirmed by NMR and HPLC-MS-ELSD at AnalytiCon Discovery) 100% (all supporting information on the compounds is available on file with AnalytiCon Discovery).

The compounds were dissolved separately in DMSO preparing 1 mM stock solutions, which were stored at 4°C until further use (maximum concentration of DMSO in 1 mM stock solutions was 0.5%). Fresh dilutions of the solutions were made from the stock solutions in CCM at the time of use for the experiments.

### Cell Survival and Short-Term Growth Assays

Cell survival was determined by using the MTT assay, which measures the mitochondrial metabolic activity, as described earlier (Mytych et al., 2016; Sodagam et al., 2017). Early passage PCS cells (p27) were used for this analysis. Furthermore, in order to rule out any interfering effects of the solvent DMSO, a set of pilot experiments were performed with equivalent concentrations of DMSO (maximum concentration 0.1%). No effects of DMSO were detected (data not shown), and therefore DMSO controls were omitted from further experiments.

Each compound was tested individually. PCS cells were exposed to various concentrations of the compound in one of the following manners: (1) appropriate volumes of the test solution were added 24 h after seeding the cells so that the cells were already attached to the growth surface of the plastic plates (for experiments on the short-term effects) or (2) appropriate volumes of the test solution were added immediately at the time of cell splitting during serial passaging for life-long experiments. Furthermore, every time the cell culture medium was replaced (mostly on a once-a-week basis) during serial passaging, the test compounds were also replaced by fresh additions. A 20,000-fold range of concentrations of individual test chemicals (from 0.01 to 200 μM) were tested. After 24 h of exposure, the MTT assay was performed on at least six wells at each concentration, and the data were presented as % MTT activity relative to the controls (Mytych et al., 2016; Sodagam et al., 2017).

Short-term growth analysis was performed as described earlier (Demirovic and Rattan, 2011; Joergensen and Rattan, 2014). Briefly, PCS cells at p14 were seeded in several 24-well plates at a density of 1 × 10^4^ cells/well. Four selected doses of individual test chemicals were added 24 h after cell seeding, and the number of cells were counted after different days (1, 3, 5, and 7) of treatment in at least two wells, and the third well in each case was fixed in ice cold methanol, stained with Giemsa stain, and photographs of the stained cells were taken under a microscope for morphological comparisons.

### Long-Term Survival, Longevity, and Aging Studies

As described earlier (Sodagam et al., 2017), life-long serial passaging of PCS cells was performed in T_75_ flasks with or without the presence of the test chemical (0.5 and 1.0 μM). During serial passaging, fresh complete medium with or without test chemicals was replaced once a week. Cells were considered to become irreversibly senescent after the cessation of cell growth during a period of more than 1 month, and when more than 90% cells were positive for senescence-specific β-galactosidase staining (Dimri et al., 1995). For telomere length and modified nucleotide level determination, PCS cells were collected as cell pellets from various passage levels, as described earlier (Mytych et al., 2016; Sodagam et al., 2017).

### Ethanol Stress Tolerance

PCS cells (p17, p33, and p55) cultured continuously with or without the test compounds were seeded at a density of 8,000 cells per well in 96-well plates in normal CCM. After 24 h, fresh medium containing 10% ethanol was added, and the cells were incubated for 1 h at 37°C, followed by MTT assay (Sodagam et al., 2017).

### Telomere Restriction Fragment (TRF) Length (Southern Blot Analysis)

Mean TRF length was measured using the TeloTAGGG telomere length assay kit (Roche Molecular Biochemical, Indianapolis, USA) with minor modifications (Sodagam et al., 2017). The total amount of DNA was measured using a freeware 1D gel electrophoresis image analysis software GelAnalyzer,^3^ and the mean TRF was calculated according to the manufacturer’s instructions. Mean TRF length was normalized to appropriate controls at p15.

### Global DNA Modifications

For evaluation of 5-methylcytosine (5-mC) and 5-hydroxymethylcytosine (5-hmC) levels in total DNA, MethylFlash Global DNA Methylation (5-mC) ELISA Easy Kit (Colorimetric, P-1030) (Epigentek, Farmingdale, NY, USA) was used according to the manufacturer’s instructions. Briefly, 100 ng DNA extracted from PCS cells was analyzed for 5-mC content. Tecan Infinite^®^ M200 absorbance mode microplate reader was used to read absorbance at 450 nm, and the calculation was made on the basis of standard curve generated with the standard controls, and presented as 5-mC/total DNA and 5-hmC/total DNA, respectively (Sodagam et al., 2017).

For evaluation of 8-hydroxy-2′-deoxyguanosine (8-OHdG, 8-oxo-dG) levels in total DNA, EpiQuik 8-OHdG DNA Damage Quantification Direct Kit (Colorimetric, P-6003) (Epigentek, Farmingdale, NY, USA) was used according to the manufacturer’s instructions. DNA from PCS cells (300 ng) was subjected to 8-OHdG content analysis, and the absorbance was read at 450 nm using the microplate reader. The calculation was made on the basis of standard curve generated with 8-OHdG standard control and the data were presented as 8-OHdG/total DNA (%).

### Statistical Analysis

The differences between the levels of 5 m-C, 5-hmC, and 8-OHdG were assessed by one-way ANOVA using GraphPad Prism 5, and with the Dunnett’s multiple comparison test. The results represent the mean ± SD or SEM. Student’s *t*-test was employed to compare the differences between dose response of PCS control cells versus PCS cells exposed to test compounds, and the differences between ethanol stress tolerance of PCS cells with or without chronic exposure to test compounds.

## Results and Discussion

### Short-Term Effects of ROSM, AMPEL, and AMOR on Cell Survival and Growth

**Figure 2:**
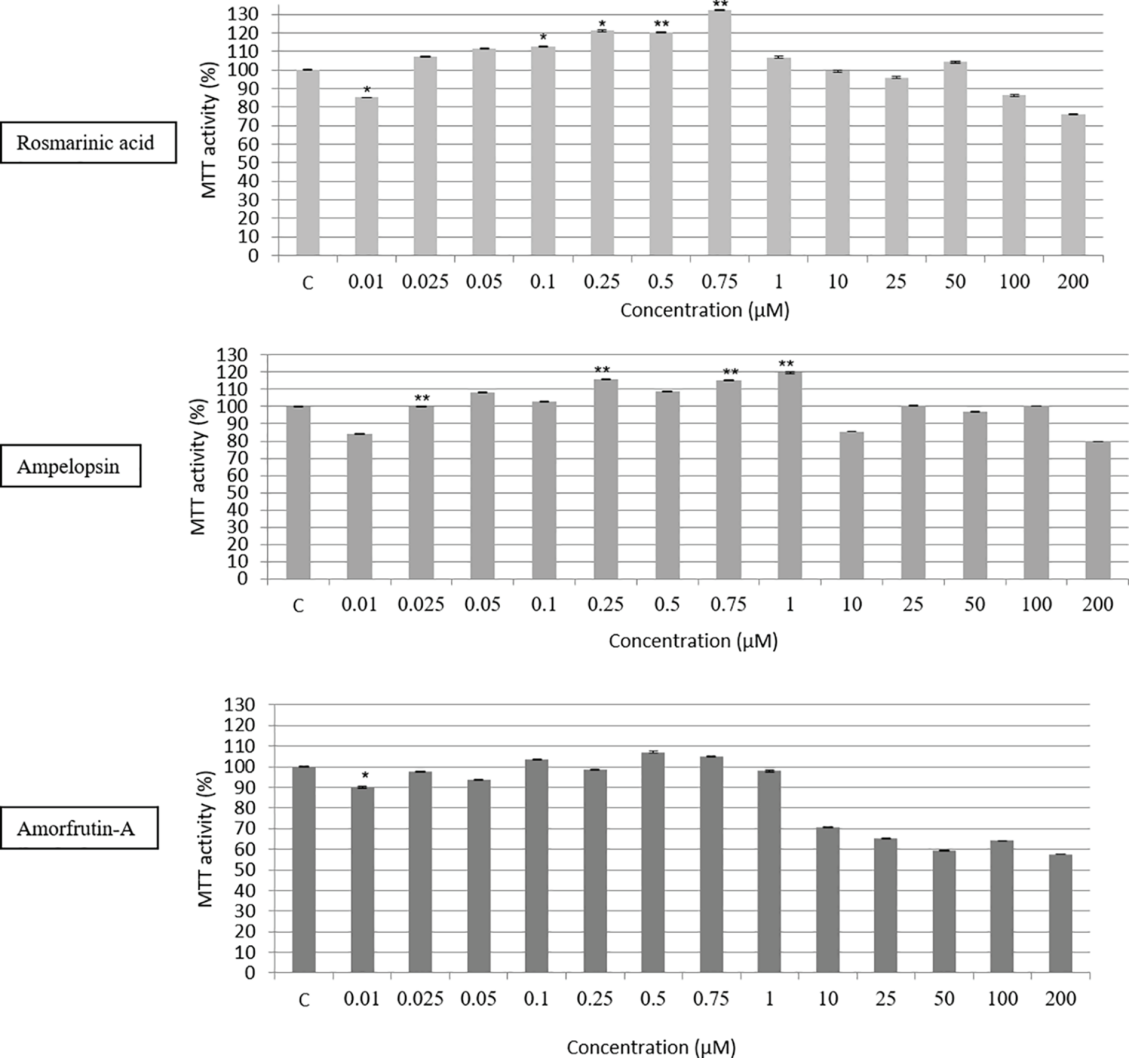
Dose response testing (between 0.01 and 200 μM) of ROSM, AMPEL, and AMOR on the metabolic activity and survival of early passage PCS cells (p17), as determined by the MTT assay. The bars indicate the ±SD; *n* = 6, in terms of independent wells; **p < 0.01, *p < 0.05, as determined by Student’s *t*-test.

A 20,000-fold concentration range (between 0.01 and 200 μM) of the three compounds, tested individually, on human skin fibroblasts (PCS cells) showed a biphasic response, determined by the MTT assay measuring the survival and metabolic activity of cells (Figure 2). Except for 0.01 μM concentration of test compounds, a 24-h exposure to any of the three compounds showed 10–30% increase in MTT activity until 0.75 or 1 μM at best, followed by a progressive inhibition of the activity in the remaining range of concentrations. The reasons for the negative effects of 0.01 μM concentration are not clear, and may reflect the nature of the MTT assay, which does not distinguish between cytostatic effects in terms of reduced metabolic activity versus cytotoxic effects, as discussed by Wang et al. (2010). Therefore, although the effect of 0.01 μM seems to be statistically significant, it does not seem to be biologically significant in the sense that there was no negative effect on the growth and proliferation of PCS cells over a relatively longer period, as described below. Further studies are required to resolve this apparently paradoxical observation. It should also be pointed out that there were some individual differences among the effects of the three compounds. For example, whereas ROSM and AMOR were most stimulatory at 0.5 and 0.75 μM doses before beginning to show their inhibitory effects, the stimulatory effects of AMPEL could be observed until 1 μM dose.

**Figure 3:**
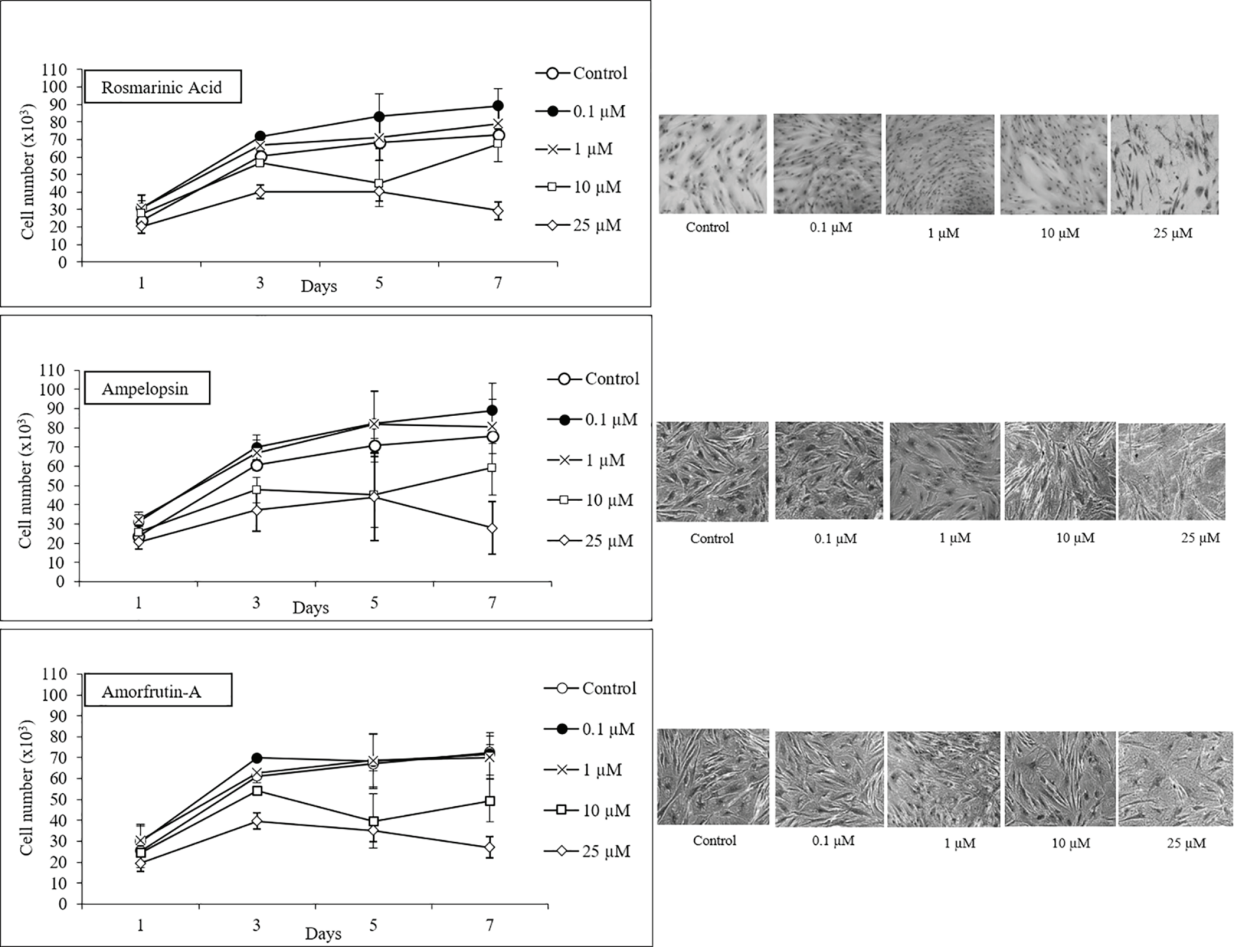
One-step growth curves and cell morphology of early passage PCS cells (p20) exposed to different concentrations of test compounds over a period of 7 days; *n* = 3; ±SEM; and photographs of Giemsa stained cells at microscopic magnification 10×.

In order to confirm the biphasic dose responses of ROSM, AMPEL, and AMOR one-step growth analysis was performed at four concentrations of each compound over a period of 7 days. Figure 3 shows that while 0.1 and 1 μM doses were generally growth supportive or even somewhat stimulatory, the concentrations above 10 and 25 μM were growth inhibitory for early passage young PCS cells. This effect is further documented by showing the microphotographs of Giemsa-stained PCS cells exposed to different concentrations of test compounds for 7 days (Figure 3). It is clear that whereas PCS cells exposed to lower doses (0.1 and 1 μM) had somewhat stimulatory and morphology-maintaining effects without any obvious negative effects, higher doses (10 and 25 μM) caused some enlargement in cell size and a reduction in the number of cells as compared with the control cells.

### Long-Term Studies of ROSM, AMPEL, and AMOR on Replicative Lifespan

Based on the short-term studies performed with a wide range of concentrations of three compounds, two concentrations (0.5 and 1 μM) of each compound were selected for long-term studies. PCS cells, serially passaged in the presence or absence of one of the three compounds, were monitored for cell survival, population doubling times, cell morphology, final CPD achieved, TRF, DNA oxidative damage level, global DNA methylation level, and ethanol stress tolerance over a period of more than 325 days. Collectively, the results showed that PCS cells could be grown throughout their lifespan *in vitro* without any obvious negative effects, and that there were some health-promoting and aging-modulatory effects as discussed below.

**Figure 4:**
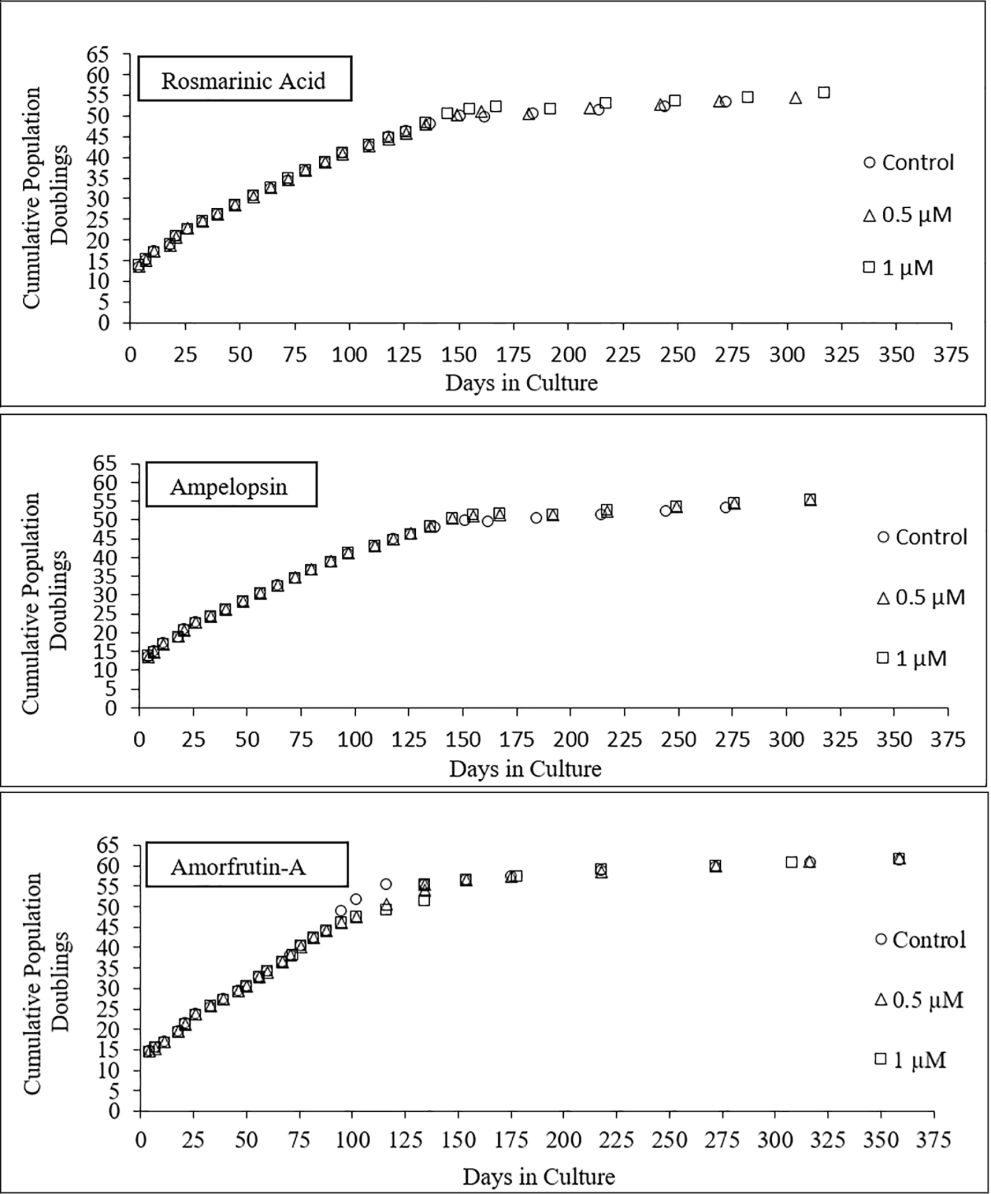
Longevity curves of PCS cells serially passaged from PDL13 (p13) onward until the end of their replicative lifespan *in vitro*, in the presence or absence of two doses of individual test compounds.

Figure 4 shows the longevity curves for PCS cells, which were serially passaged from PD13 onward in the absence (control) or presence of 0.5 and 1 μM ROSM, AMPEL, and AMOR (Figure 4) until the end of their replicative lifespan. During the first part of life and until about CPDL50 attained in about 150 days, there were no differences in the growth rates, cell yield, and general morphology of treated and untreated cells. From that point onward, and until the end of their replicative lifespan *in vitro*, the treated cells seem to have some advantage as compared with the untreated cells in terms of faster growth rates and higher cell yield. It should be pointed out that in the case of AMOR-treated cells, there was a temporary interruption in their continuous growth at PD50. Therefore, an ampoule of frozen PCS at an equivalent PDL was thawed and the remaining part of serial passaging was completed in the presence or absence of AMOR. Eventually, untreated control cells become fully senescent at CPDL57, which was one or two PDs earlier than the treated cells, during a total period of 325–360 days. Therefore, no claims can be made for any significant effects on the replicative lifespan of PCS cells exposed to any of the three compounds.

### ROSM, AMPEL, and AMOR-Mediated Changes in Telomere Length

**Figure 5:**
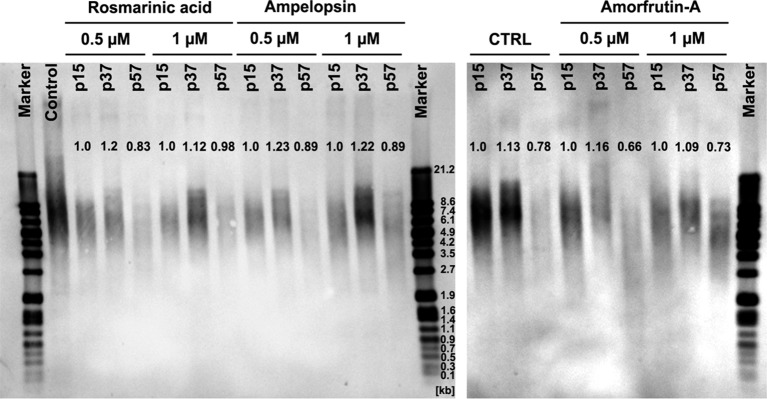
Telomere restriction fragment (TRF) length, by Southern blot analysis, in serially passaged PCS cells at different passage levels, with and without continuous exposure to selected concentrations of test compounds. Mean TRF was calculated using GelAnalyzer software and normalized to appropriate controls at p15.

A progressive loss of telomeres and shortening of telomere restriction fragment (TRF) length is one of the well-established molecular markers of aging of cells *in vitro* and *in vivo* (Mather et al., 2011; Jäger and Walter, 2016; Lopez-Otin et al., 2016). Figure 5 shows that serially passaged untreated control PCS cells at senescence (p57) had about 22% shorter TRF as compared with early passage young cells (p15). In comparison, senescent cells which were grown in the chronic presence of either ROSM or AMPEL did not lose TRF to the same extent as the control cells (between 5 and 10% less loss), but it was not a complete prevention. However, in the case of AMOR, even this much preventive effect could not be observed. It should also be pointed out that the middle-aged cells (p37) under all conditions had somewhat longer telomeres as compared with early passage (p15) cells. The significance of these results with respect to longer TRF in middle age is not clear and may reflect a general cell culture status-related variation. What, however, is clear that ROSM and AMPEL do slow down the rate of telomere loss to some extent during replicative senescence of PCS cells. A possible explanation for the phytochemical-mediated effects on telomere length may be due to their ability to target several functions of telomeres not necessarily associated with telomerase activity. For example, this may affect the functions of proteins of shelterin complex as have already been documented for two dietary flavones, acacetin and chrysin (Boussouar et al., 2013). Furthermore, our observations concur with the report that senescent human skin fibroblasts have markedly reduced levels of TERT protein and telomerase activity, suggesting the importance of the non-canonical (telomere-unrelated) functions of TERT in cellular senescence (Yehuda et al., 2017).

### ROSM, AMPEL, and AMOR-Induced Global DNA Modifications

**Figure 6:**
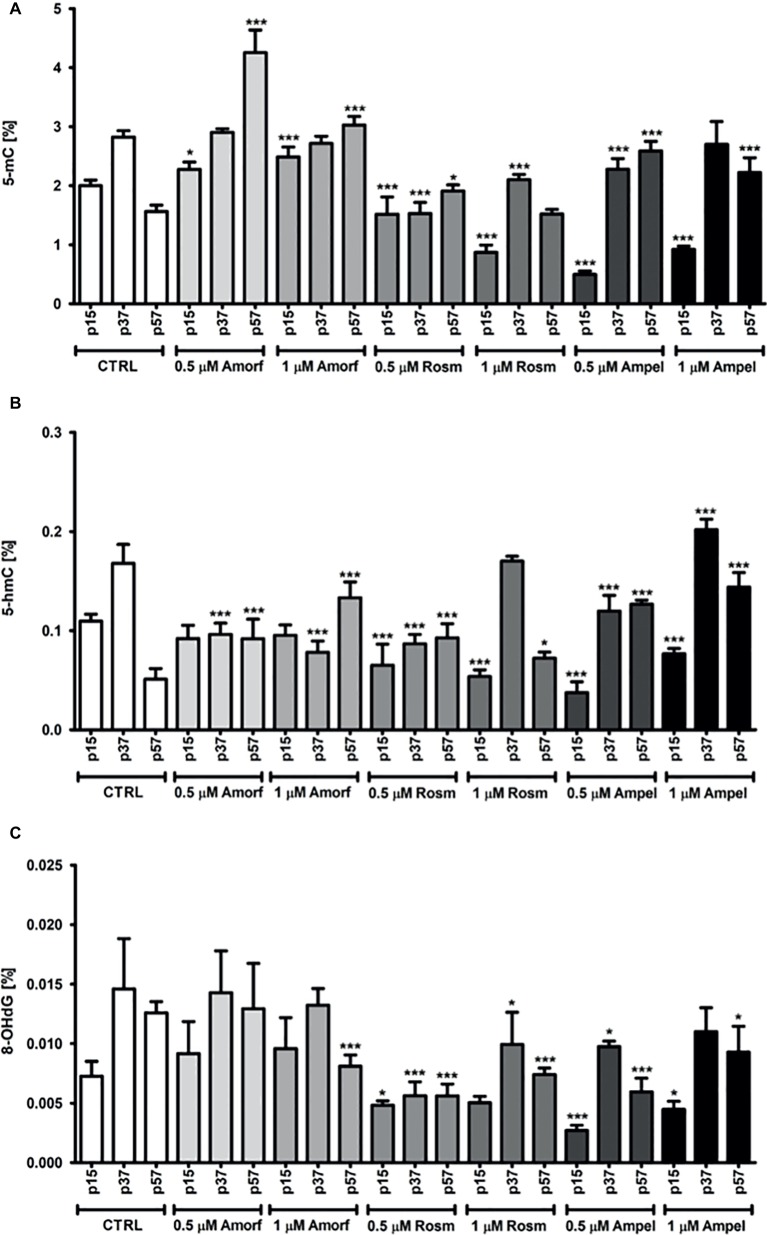
Effect of continuous exposure to test compounds on the levels of 5-mC **(A)**, 5-hmC **(B)**, and 8-OHdG **(C)** in DNA isolated from serially passaged PCS cells. The bars indicate the SEM, *n* = 3, ***p < 0.001, *p < 0.05 compared to passages, p15, p37, and p57, at control growth conditions, respectively (ANOVA and Dunnett’s *a posteriori* test).

We have also measured other epigenetic molecular markers of cellular aging, 5-mC, 5-hmC, and oxidative stress marker 8-OHdG (Wolf et al., 2002; Rattan, 2008, 2018; Lopez et al., 2017) in the DNA extracted from PCS cells with or without exposure to one of the three tested phytochemicals. Figure 6 shows that, as expected, there was a significant decrease (about 33%) in the levels of 5-mC in the control untreated cells during passaging (Figure 6A). Interestingly, at early passage (p15), both ROSM and AMPEL acted as DNA hypomethylating agents reducing the 5-mC levels by 56–75%, respectively (Figure 6A). However, a life-long exposure to these compounds lead to the maintenance of relatively higher levels of 5-mC in late passage (p57) senescent cells as compared to the controls. The levels of the product of 5-mC oxidation, namely 5-hydroxymethylcytosine (5-hmC) were also analyzed, and similar changes in the levels of 5-hmC were observed in ROSM and AMPEL-treated cells (Figure 6B). By contrast, AMOR treatment increased the levels of 5-mC at all passages, but did not generally affect the levels of 5-hmC. It is widely accepted that 5-hmC is in fact a *bona fide* epigenetic mark with a key role in epigenetic reprogramming during development, aging, cancer, and neurodegenerative disorders, and not a mere transient molecule in DNA demethylation pathway (Lopez et al., 2017). However, the underlying mechanisms are still largely unknown and, therefore, it is an important issue to determine the possible function role of 5-hmC in these processes.

We have also measured the levels of one of the oxidative DNA damage-products, namely 8-hydroxy-2′-deoxyguanosine (8-OHdG, 8-oxo-dG) in the phytochemical-treated and untreated PCS cells (Figure 6C). Whereas both ROSM and AMPEL significantly protected DNA from oxidation during passaging, AMOR provided only some protection at 1 μM seen best in the late passage cells (Figure 6C).

### Ethanol Stress Tolerance

**Figure 7:**
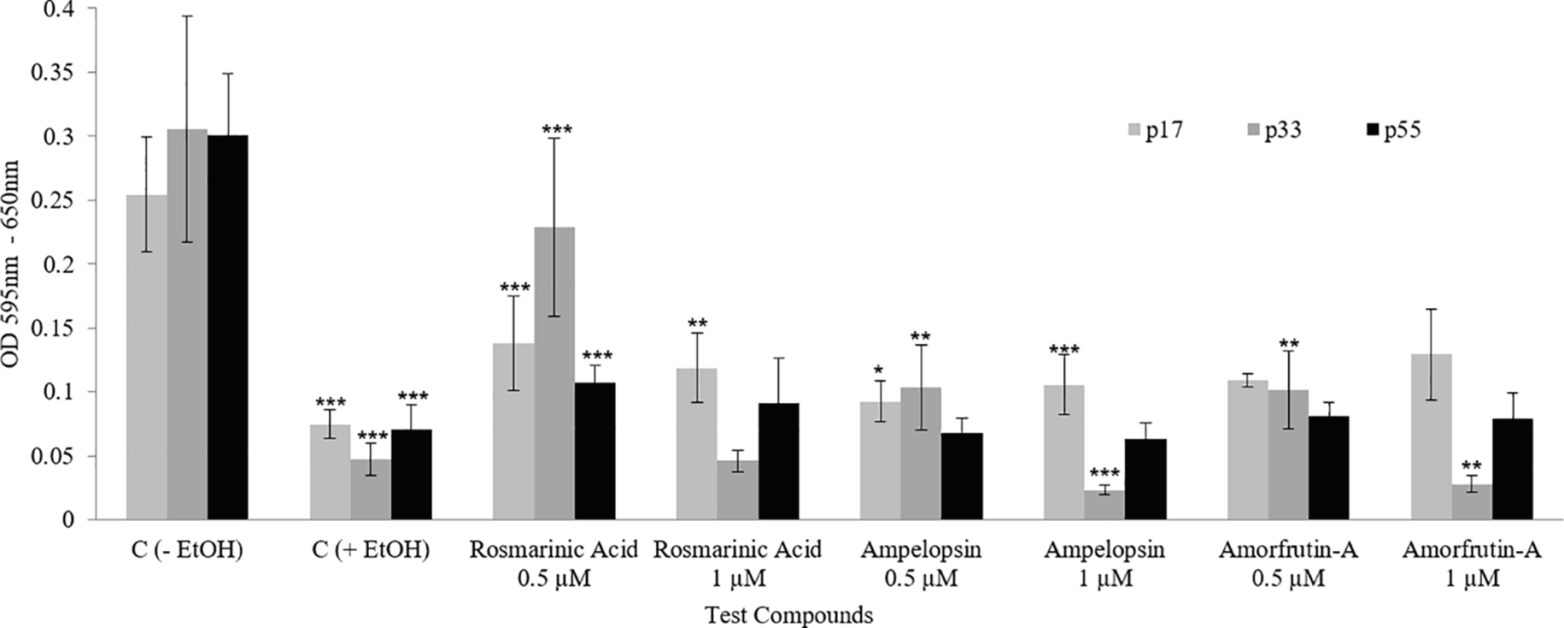
Ethanol stress tolerance of PCS cells at three ages (p17, p33, and p55) of serially passaged PCS cells with or without continuous exposure to selected doses of test compounds, as determined by the MTT assay. The bars indicate the ±SD; *n* = 6, in terms of independent wells; ***p < 0.001, **p < 0.01, *p < 0.05; as determined by Student’s *t*-test.

As previously mentioned, “a functional test for demonstrating the health beneficial effects of a treatment is to compare the stress tolerance ability of cells, which is a crucial aspect of cellular homeodynamics and homeodynamic space” (Sodagam et al., 2017; Rattan, 2018). Figure 7 shows that a chronic exposure of PCS cells to individual phytochemicals resulted in an increased ability to tolerate ethanol stress, as determined by the MTT assay. Phytochemical-treated PCS cells had significantly increased resistance toward ethanol-induced reduction in the mitochondrial MTT activity at all tested ages. For example, the untreated control cells were very sensitive to the inhibitory effects of 10% ethanol (between 80 and 90% inhibition), whereas treated cells had 20–44% more resistance to ethanol (Figure 7). A comparison among the three compounds shows that ROSM was the most effective compound, followed by AMOR and AMPEL, respectively, with respect to their effects in improving the stress tolerance levels of PCS cells.

## Conclusions and Future Perspective

The results of this series of studies show that the three phytochemicals, ROSM, AMPEL, and AMOR, selected from the ongoing Horizon2020 project AwE (Luyten et al., 2016) have some modulatory effects on human skin fibroblasts. These effects are not very dramatic, but they do indicate that these compounds individually have some beneficial effects in terms of supporting the long-term growth, and affecting the rate of loss of telomeres, 5-mC and 5-hmC. Furthermore, these phytochemicals also seem to reduce the accumulation of oxidative DNA damage product 8-OHdG and improve the stress tolerance ability.

At this state, it is not clear as to what are the exact mechanisms of action of these compounds. One possibility is that these polyphenols and flavonoids could be potential hormetins in the sense that they induce one or more stress response pathways in the cells (Rattan, 2012; Govindan et al., 2018; Gurau et al., 2018). Indeed, our preliminary studies show that ROSM, AMPEL, and AMOR induce heat shock response by stimulating the synthesis of heat shock protein (Hsp70) by more than two-fold in early passage young PCS cells (results not shown). This is in agreement with our earlier reports on the hormetic nature of kinetin, curcumin, and rapamycin by virtue of their ability to induce one or more stress responses in human cells (Berge et al., 2008; Demirovic and Rattan, 2011; Lima et al., 2011; Sodagam et al., 2017).

We do realize that these observations need to be extended further and detailed studies should be performed. One important study, now in progress in our labs, is by combining the tested compounds and determining their short-term and long-term effects on various biological and molecular markers of aging of human cells. The initial results obtained after more than 100 days of continuous exposure to different combinations of ROSM, AMPEL, and AMOR indicate possible additive or synergistic health beneficial effects. Such studies combining more than one compound for testing their effects on health, health-span, aging, and longevity are nearer to the daily life reality of food consumption, rather than a purely reductionistic approach of testing one nutritional component at a time.

## Author Contributions

LS performed all the cell biology experiments. AL, EK, and SK performed molecular markers analyses. KS provided the test materials and reviewed the writing. MW analyzed the molecular data and helped in writing. SR designed and supervised the study, and wrote the paper.

### Conflict of Interest Statement

KS was employed by the company AnalytiCon Discovery GmbH, Potsdam, Germany.

The remaining authors declare that the research was conducted in the absence of any commercial or financial relationships that could be construed as a potential conflict of interest.

## Footnotes

^1^https://ec.europa.eu/research/participants/portal/desktop/en/opportunities/h2020/topics/693-phc-01-2014.html

^2^http://www.h2020awe.eu/

^3^www.gelanalyzer.com
